# Faecal metabarcoding provides improved detection and taxonomic resolution for non-invasive monitoring of gastrointestinal nematode parasites in wild moose populations

**DOI:** 10.1186/s13071-022-05644-6

**Published:** 2023-01-18

**Authors:** Marie L. Davey, Stefaniya Kamenova, Frode Fossøy, Erling J. Solberg, Rebecca Davidson, Atle Mysterud, Christer M. Rolandsen

**Affiliations:** 1 Norwegian Institute for Nature Research (NINA), Trondheim, Norway; 2 University of Oslo, Oslo, Norway; 3 Norwegian University of Life Sciences, Ås, Norway; 4 Norwegian Veterinary Institute, Tromsø, Norway

**Keywords:** Nemabiome, Metabarcoding, ITS2, DNA extraction method, NC1–NC2 primers, *Alcesalces*, Helminth, Ungulates

## Abstract

**Background:**

Although wild ungulate populations are heavily monitored throughout Europe, we understand little of how parasites affect population dynamics, and there is no systematic, long-term monitoring of parasite diversity and parasite loads. Such monitoring is in part hampered by a lack of time- and cost-effective assay methodologies with high sensitivity and good taxonomic resolution. DNA metabarcoding has been successfully used to characterize the parasitic nemabiome with high taxonomic resolution in a variety of wild and domestic hosts. However, in order to implement this technique in large-scale, potentially non-invasive monitoring of gastrointestinal parasitic nematodes (GIN), protocol optimization is required to maximize biodiversity detection, whilst maintaining time- and cost-effectiveness.

**Methods:**

Faecal samples were collected from a wild moose population and GIN communities were characterized and quantified using both parasitological techniques (egg and larva counting) and DNA metabarcoding of the ITS2 region of rDNA. Three different isolation methods were compared that differed in the volume of starting material and cell lysis method.

**Results:**

Similar nematode faunas were recovered from all samples using both parasitological and metabarcoding methods, and the approaches were largely congruent. However, metabarcoding assays showed better taxonomic resolution and slightly higher sensitivity than egg and larvae counts. The metabarcoding was not strictly quantitative, but the proportion of target nematode sequences recovered was correlated with the parasitologically determined parasite load. Species detection rates in the metabarcoding assays were maximized using a DNA isolation method that included mechanical cell disruption and maximized the starting material volume.

**Conclusions:**

DNA metabarcoding is a promising technique for the non-invasive, large-scale monitoring of parasitic GINs in wild ungulate populations, owing to its high taxonomic resolution, increased assay sensitivity, and time- and cost-effectiveness. Although metabarcoding is not a strictly quantitative method, it may nonetheless be possible to create a management- and conservation-relevant index for the host parasite load from this data. To optimize the detection rates and time- and cost-effectiveness of metabarcoding assays, we recommend choosing a DNA isolation method that involves mechanical cell disruption and maximizes the starting material volume.

**Graphical Abstract:**

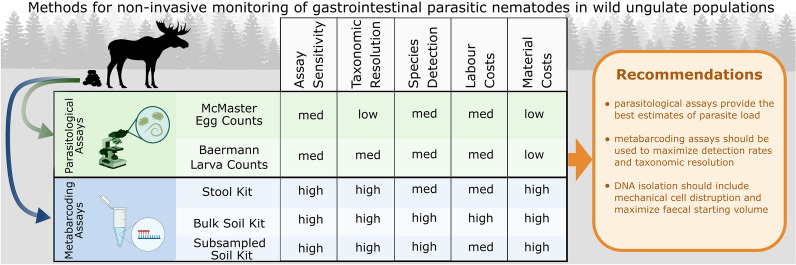

**Supplementary Information:**

The online version contains supplementary material available at 10.1186/s13071-022-05644-6.

## Background

Ungulates are an economically and culturally important group of species in Europe [1], with a current estimated annual harvest of above 7 million individuals [2]. The population ecology of ungulates is well-described in terms of how population density and climate affect vital rates [3, 4], yet we have a limited understanding of the role of parasites in population limitation and regulation. There is some evidence of negative impacts of parasites on host fitness, including body condition, survival, and fecundity [5–8], but in a very limited set of species and countries. Moreover, the few studies quantifying parasites in wild ungulates are typically short-term [5, 6, 9, 10]. Long-term monitoring of ungulate populations in Europe is extensive and uses either direct estimation of abundances and body condition, or indirect monitoring of browsing pressure on important forage species [11–13]. However, there is no long-term monitoring of parasite diversity and parasite loads in wild ungulates in Europe, in part due to a lack of suitable methods for estimating parasite diversity and abundance, which are required for efficient monitoring.

Parasite monitoring of gastrointestinal nematode (GIN) communities in wild ungulate populations is methodologically challenging. Traditional parasitological methods for assessing GINs can be labour-intensive and not well suited to large-scale, non-invasive, long-term monitoring. Species-level identification of GINs typically requires adult specimens. This necessitates harvesting gastrointestinal material from individuals that have been hunted, culled, lethal sampled, or died of natural causes [6, 14, 15]. This makes it challenging to have systematic population representative sampling and non-invasive monitoring in wild populations. Faecal egg counts are frequently used to measure gastrointestinal helminth burden in livestock (e.g. [7, 16]). However, egg counting requires considerable effort, training, and taxonomic expertise, making this task quite demanding for use on large numbers of samples. Gastrointestinal nematodes belonging to the order Strongylida produce eggs that are morphologically similar and identification therefore requires either molecular identification of the eggs or morphological speciation of hatched larvae, after culture of the eggs and larvae. This includes a variety of species, from *Ostertagia* sp. (superfamily Trichostrongyloidea) in the abomasum to *Bunostomum* sp. (superfamily Ancylostomatoidea) in the small intestine to *Chabertia* sp. (superfamily Strongyloidea) in the large intestine [17]. The taxonomic resolution of egg count surveys is subsequently low, and groups together organisms that can have different or interactive impacts on their hosts [18].

The molecular characterization of GIN parasites offers methodological alternatives to traditional parasitological approaches. A variety of PCR-based methods detect, identify, and quantify GIN species in research and diagnostic settings [19, 20]. The primary advantage of molecular approaches in the characterization of GIN parasites has been the reliable identification of these species at any life stage [19, 21]. Recently, both free-living and parasitic nemabiome diversity has been investigated in a variety of environments and hosts using DNA metabarcoding techniques that rely on high-throughput sequencing methods (e.g. [22–26]). In particular, DNA metabarcoding of the internal transcribed spacer 2 (ITS2) region of ribosomal DNA (rDNA) specifically targeting clade V parasitic GIN has been successfully applied to adult worms, eggs, and faecal samples (e.g. [27–29]). Combined with a curated, well-developed reference sequence database (www.nemabiome.ca), this provides high quality data with good taxonomic resolution for parasitic GIN communities. This method has been used to successfully characterize the GIN communities hosted by a variety of wild ungulates [27, 28, 30, 31] and shows substantial promise for allowing non-invasive monitoring of GINs in wild populations [31].

In order to implement DNA metabarcoding in large-scale, potentially non-invasive monitoring of GIN, protocol optimization is required to maximize biodiversity detection, whilst maintaining time- and cost-effectiveness in the protocol. The DNA extraction method has been documented to impact the recovery of soil nematode biodiversity [32]. More specifically, the detection and sensitivity of polymerase chain reaction (PCR)-based assays for GIN from faecal samples vary with the type of DNA extraction method used [33, 34]. However, the impact of the DNA extraction method on parasitic GINs recovery specifically using DNA metabarcoding of faecal samples has not been previously examined, either in terms of biodiversity recovery or in terms of time- and cost-effectiveness. Typically, commercial kits for DNA extraction from faecal material are optimized to retrieve high-quality DNA from a large range of target organisms from both fresh and frozen material. However, they rely on small volumes of starting material, thus potentially limiting the capacity to capture sporadic DNA from gastrointestinal parasites, especially at periods of low egg-shed. On the other hand, DNA extraction kits from soil provide similar purification steps for removal of inhibitory compounds while accommodating large volumes of starting material [35, 36]. However, using them is comparatively cost- and time-laborious, potentially negating their advantages in the context of large-scale monitoring programs.

Here, we assess the impact of the DNA isolation method from frozen faecal samples on the results of ITS2 DNA metabarcoding of clade V GIN communities with the aim of contributing to a robust protocol suitable for routine studies and long-term parasite monitoring in wild ungulate populations. Using faecal samples collected during the capture and global positioning system (GPS)-collaring of moose (*Alces alces*), we compare the results of metabarcoding inventories using commercially available DNA isolation kits that differ in the (i) amount of starting material, (ii) method of cell disruption and (iii) labour required. The results were compared with those from traditional methods, in our case standard egg (McMaster) and larvae (Baermann) counting, with a focus on the detection of GIN diversity and the potential for quantification of GIN parasite load, as well as the time- and cost-effectiveness that must be considered for methods to be effective and practical in large-scale monitoring practices.

## Methods

### Study area

**Fig. 1:**
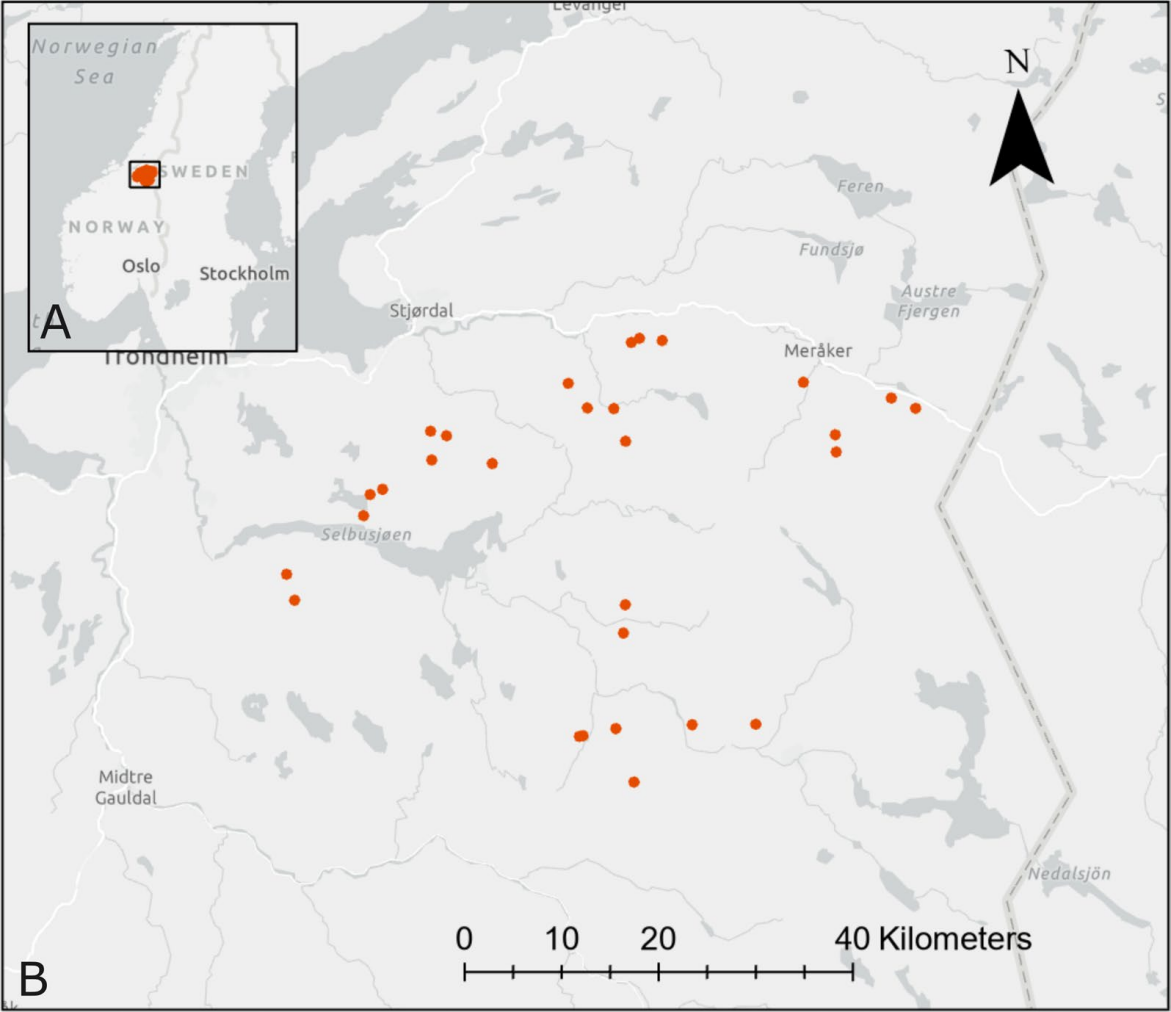
Map of study area. Maps showing (**a**) the location of the study area in central Norway and (**b**) an overview of the study area with points representing the locations where faecal samples were collected from 29 GPS-collared moose

The study area is located in Trøndelag county in central Norway within the boreal and alpine vegetation zones (Fig. 1). The vegetation is dominated by Scots pine (*Pinus sylvestris*), Norway spruce (*Picea abies*), and downy birch (*Betula pubescens*), with grey alder (*Alnus incana*), aspen (*Populus tremula*), rowan (*Sorbus aucuparia*), and goat willow (*Salix caprea*) also commonly occurring [37]. The study area spans a gentle elevational gradient between approximately 200–700 m above sea level, with active agricultural lands primarily occupying valley bottoms and the lower-lying parts of the study area.

### Sample collection

All faecal samples were collected fresh directly from the rectum of moose that had been captured and anesthetized to equip them with GPS collars to study their space use (Rolandsen et al., unpublished data). Moose were darted from a helicopter during winter, and all procedures were approved by the Norwegian Environment Agency and the Norwegian Food Safety Authority, which is the animal research authority in Norway. Twenty-nine individuals were sampled, and no recaptures were included in the analyses. Five to 10 faecal pellets were selected and placed in individual clean plastic containers. Faecal samples were sent at ambient temperatures for parasitological analyses (McMaster and Baermann counts), and thereafter stored at −20 °C until DNA extraction for metabarcoding analysis.

### Nematode counts and identification

The abundance of endoparasitic eggs and oocysts was estimated using a modified McMasters method and zinc–chloride/sodium chloride flotation fluid (with a specific gravity of 1.3) [17, 38] with a 3 g faecal sample mixed with 57 ml tap water. A total of 1 ml flotation fluid was examined for eggs giving a theoretical detection limit of 20 eggs per gram (EPG)/oocysts per gram (OPG). Eggs and oocysts were identified to genus level (*Moniezia* sp., *Trichuris* sp., *Nematodirus* sp., and *Eimeria* sp.) and, where possible, species level (*Strongyloides* *papillosus*, *Nematodirus battus*), based on morphological characteristics. Several GIN eggs can only be identified to order, given morphological similarities and size overlap. Therefore, *Chabertia* sp., *Cooperia* sp., *Haemonchus* sp., *Oesophagostomum* sp., *Ostertagia* sp., *Spiculopteragia* sp., *Teladorsagia* sp. and *Trichostrongylus* sp. were grouped as strongyle-type eggs. The Baermann technique was used to isolate, quantify and identify parasitic first-stage (L1) larvae in the faeces [38]. A 10-g faecal sample, wrapped in gauze, was suspended in tepid water in a conical glass for a minimum of 12 h at room temperature. The fluid above the 10 ml mark was aspirated and discarded, whilst the bottom 10 ml, including the sediment, was transferred to a 15 ml conical tube and centrifuged at 1500×*g* for 5 min. The supernatant was then aspirated to the 1 ml mark and a 100-μl subsample of remaining homogenized sediment examined at ×100 magnification for larvae. Larvae were identified and counted. Larvae were recorded as hatched GIN larvae, the lungworm *Dictyocaulus* sp. or dorsal spine larvae (DSL, protostrongylid larvae) based on the morphological appearance of the tail (straight tail/s-shaped tail with spine) as well as larval length. Only the first 10 protostrongylid larvae in each sample were measured to evaluate whether the animal had a mono- or mixed infections with protostrongylid larvae. The number of larvae per gram faeces (LPG) was estimated from the subsample count (number of larvae detected in 100 μl × 10/the weight of the faeces in the faecal sample). A second 100-μl subsample was taken from the unhomogenized sediment if no larvae were detected in the first subsample. If no larvae were detected in the second subsample, then the results were recorded as no larvae detected.

### Molecular detection of intestinal parasites

#### DNA extraction and sequencing

**Fig. 2:**
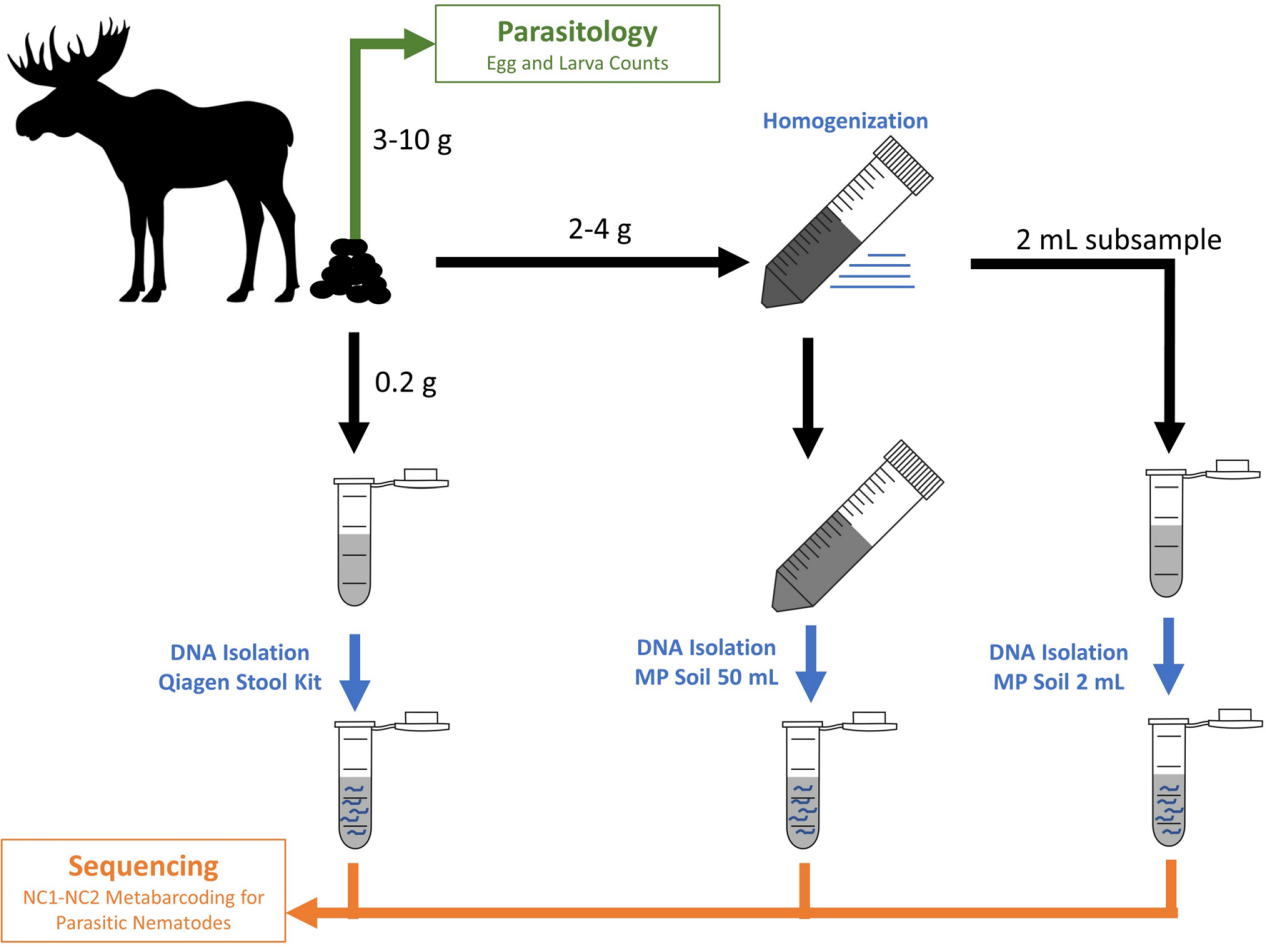
Schematic representation of the methodological comparisons investigated in this study. Faecal samples were subjected to both parasitological egg and larva counts, and subjected to three types of DNA isolation protocols and the GIN communities characterized using metabarcoding of the ITS2 region of rDNA

Faecal samples were defrosted overnight at 4 °C and each sample thoroughly homogenized in a clean zip-lock bag. DNA was isolated from subsamples of each faecal sample using one of three DNA isolation protocols (Fig. 2):

1. QIAmp Fast DNA Stool Mini Kit
  From each faecal sample, 220 mg of wet weight was withdrawn using a disposable lab spatula (Chemglass, UK). Subsamples were stored in sterile 2-ml microcentrifuge tubes and DNA was extracted immediately after sub-sampling. DNA extractions were carried out using the QIAamp Fast DNA Stool Mini Kit (Qiagen, Germany) according to the manufacturer’s instructions. Two blank extractions (ultra-pure Milli-Q water instead of DNA) were included to monitor for possible contamination.
2. MP FastDNA™ Spin Kit for Soil (50 ml volume)
  Between 1.5 and 4 g of faecal wet weight was placed in a 50-ml centrifuge tube containing Lysing Matrix E (MP Biomedicals), which comprises 1.4 mm ceramic spheres, 0.1 mm silica spheres and 25 4-mm glass beads. Samples were homogenized by shaking at 6 m/s for 40 s. DNA was isolated from the faecal material using the MP FastDNA™ Spin Kit for Soil (50 ml) according to the manufacturer’s directions, but excluding the initial three steps intended to remove humus and litter from soil samples.
3. MP FastDNA™ Spin Kit for Soil (2 ml volume)
  Two millilitres of the homogenized, lysed faecal suspension prepared in protocol 2 was transferred to a 2-ml microcentrifuge tube and DNA was isolated from it using the MP FastDNA™ Spin Kit for Soil (2 ml) according to the manufacturer’s directions, beginning the protocol with the addition of protein precipitation solution (PPS) according to the 2-ml protocol.

The NC1–NC2 primer set targeting the clade V group of parasitic GINs [39] was used to amplify the ITS2 region of rDNA from the DNA isolated from the faecal samples, isolation negative controls, and from three PCR-negative controls containing water instead of template DNA. PCR reactions contained 1× KAPA HiFi HotStart ReadyMix (Roche, Switzerland), 0.2 µM of the forward and reverse primers, and 25 ng template DNA with a final volume of 25 µl. PCR conditions consisted of an initial denaturing step of 5 min at 95 °C, followed by 35 cycles of 1 min at 95 °C, 1 min at 54 °C and 1 min at 72 °C with a final elongation step of 5 min at 72 °C. PCR products were quantified using an Agilent 4200 TapeStation and cleaned of excess primers and nucleotides using magnetic beads (Mag-Bind RxnPure Plus) to select fragments between 200 and 600 base pairs (bp) in length. The size-selected amplicons were used as a template for a second, indexing PCR using the Nextera XT Index Kit (Illumina, USA) according to the manufacturer’s instructions. The indexed samples were again cleaned as described above, pooled in equimolar amounts, and sequenced in one paired-end 300 bp run on the Illumina MiSeq sequencing platform with v3 chemistry at the Genomics Core Facility (GCF), Norwegian University of Science and Technology (NTNU), Trondheim, Norway.

#### Bioinformatics and statistical analyses

The MiSeq Reporter on the Illumina MiSeq sequencing platform was used to demultiplex samples and remove adapters. Primer sequences were identified and removed from both the 5′ and 3′ ends of forward and reverse reads using cutadapt v.1.9.1 [40], allowing up to 15% mismatch across the length of the primer. Quality filtering, error correction, and chimera detection were all conducted using the DADA2 v.1.12 package for R [41]. Reads were quality filtered to remove all sequences with ambiguous bases, > 2 expected errors in the forward direction and reverse directions, and length < 50 bp after truncation at the first instance of a base with a quality score < 15. Error rates were estimated for forward and reverse sequences and forward and reverse reads were merged with a minimum overlap of 30 bp, and amplicon sequence variants (ASVs) were inferred for each sample. Chimeric sequence variants were assessed on a per-sample basis, as chimeric events occur at the individual PCR level. If a sequence variant was flagged as chimeric in more than 90% of the samples it occurred in, it was removed. Taxonomy was assigned to ASVs using the naïve Bayesian classifier [42] implemented in DADA2 and a custom version of the Nematode ITS2 v.1.0.0 database ([24, 43], www.nemabiome.ca), including additional reference sequences of *Nematodirus,* *Nematodirella*, *Spiculopteragia*, and *Dictyocaulus* species retrieved from GenBank (Accession No: MW837830-MW837840, KT438069, AY168865). Minimum confidence estimates of 80% were required for a successful assignment against the custom database at any given taxonomic level. Each ASV was also subjected to a BLAST search against the NCBI nucleotide non-redundant database. Any ASV with the best BLAST match to a lineage outside the order Strongylida, or that could not be assigned with confidence > 80% at the order level was designated a non-target amplification and excluded from further analyses. ASVs that could not be successfully assigned to the species level were clustered using VSEARCH [44] at 97% sequence similarity to create species-unit proxies that were subsequently assigned taxonomy at the genus or family level.

All statistical analyses were conducted in the R statistical environment [45]. To examine variation in the proportional abundance of the individual taxa recovered from a sample by each method, log twofold changes between methods were calculated per pairwise sample:method combination for each taxon recovered. General linear models were used to assess differences in ASV and species recovery between DNA isolation protocols, with the log-transformed sequencing depth included as a fixed effect and biological sample included as a random effect in both cases. A general linear model with a quasibinomial distribution was used to assess the relationship between the proportion of target nematode reads recovered and the total GIN egg and larvae count per gram of faeces. The DNA isolation method was included in the model as a fixed effect.

## Results

### Parasitological assays

**Fig. 3:**
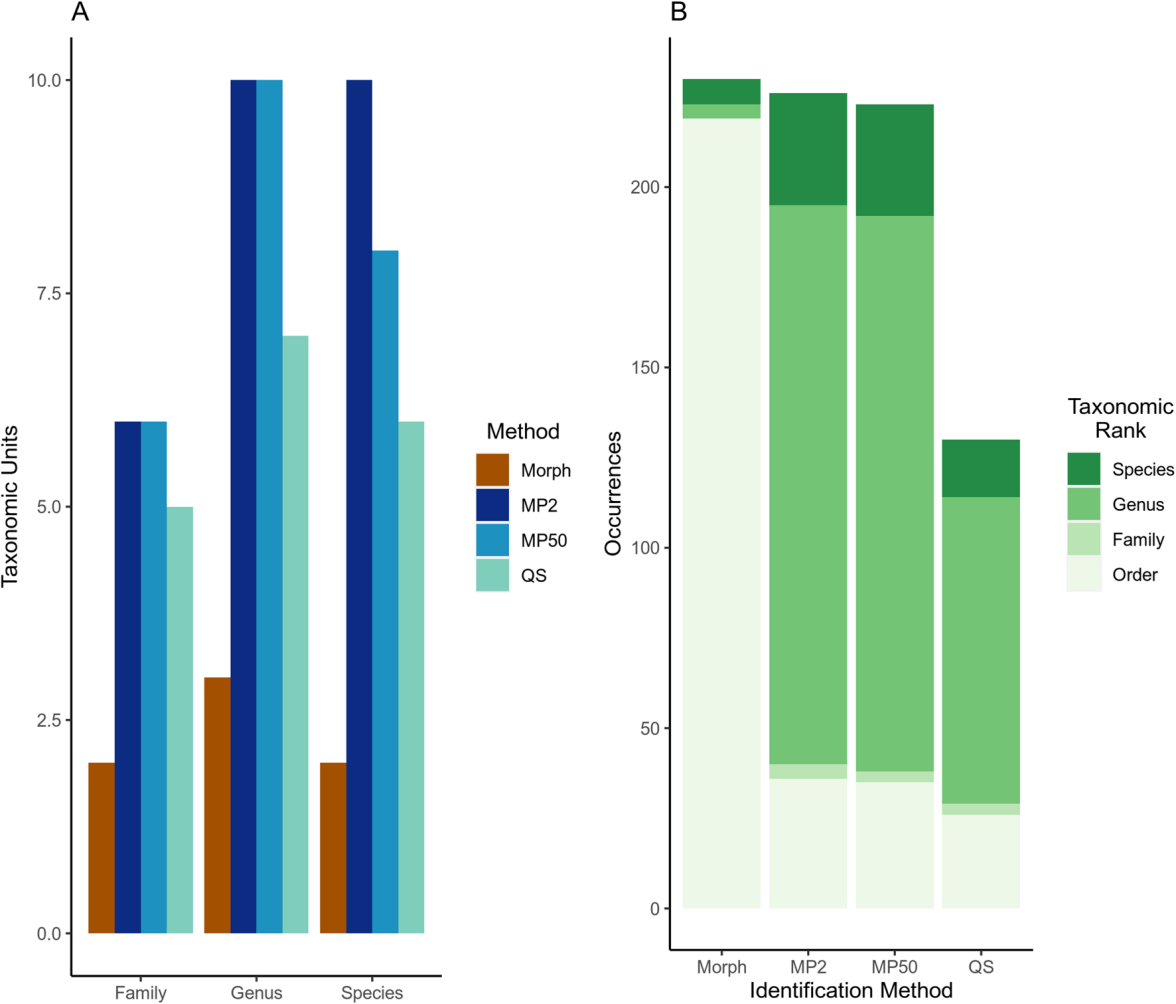
Taxonomic resolution of parasitological and molecular methods. Comparisons of the (**a**) total taxonomic diversity recovered by the different parasitological and molecular methods at the family, genus, and species levels and (**b**) the taxonomic resolution achieved for the occurrences detected by each method. In (**a**) the taxonomic units on the *y* axis represent the detected number of families, genera, and species for each of the three groupings from left to right, respectively. *Morph* morphological, *MP2* MP soil kit 2 ml, *MP50* MP soil kit 50 ml, *QS* Qiagen stool kit

Visual counts of eggs and larvae found moose hosted an average of 1.6 (± standard deviation [SD]: 1.09) nematode taxa per individual, although 7% (2/29) of individuals had no detectable parasites in their faeces (Tables 1; Additional file 2: Tables S1–S4). Strongylid-type nematodes were the most frequently detected taxon, occurring in all but three individuals. All other taxa (*Trichuris,* *Capillaria,* *Nematodirus,* *Elaphostrongylus,* *Varestrongylus)* were only detected sporadically across individuals (Table 1). Most eggs and larvae could only be identified to the order level, with the exception of *Nematodirus*, *Trichuris*, and *Capillaris* eggs, and larvae of *Varestrongylus* *alces* and *Elaphostrongylus* *alces* (Fig. 3B; Additional File 1: Figs. S1–S2). Overall, parasitological assays detected fewer unique taxonomic units than metabarcoding assays at the family, genus, and species levels (Fig. 3A). The average parasitic GIN egg load was 43.9 eggs per gram of faeces but varied up to two orders of magnitude between individuals (SD: 36.8, range: 0.140). The average parasitic GIN larval load was also highly variable, with a mean of 5.6 larvae per gram of faeces (SD: 16.6, range: 0–82.14).

**Table 1:** Gastrointestinal nematode parasite loads estimated from moose faecal samples (*n* = 29)

Taxon	No. positive individuals	EPG^b^ ± SD (range)	LPG^c^ ± SD (range)
Strongyle-type eggs	26	47.5 ± 35.8 (1:140)	NA
*Nematodirus* spp. eggs	3	13.5 ± 10.8 (1:20)	NA
*Trichuris* sp.	8	46.7 ± 44.9 (20–155.1)	NA
*Capillaria* sp.	2	19.7 ± 0.43 (19.4–20)	NA
Strongylid-type larvae	3	NA	38.51 ± 37.8 (16.3:82.1)
Protostrongylid-type larvae^d^	4 3V/4E^a^	NA	11.7 ± 10.0 (3.16:25)

Twenty-nine moose faecal samples were analysed using the McMaster and Baermann techniques. The total number of individual moose testing positive for a given group is reported, as well as the eggs or larvae per gram of faeces

*NA* not applicable

^a^V/E: number of individuals infected with *Varestrongylus* *alces*/*Elaphostrongylus* *alces*

^b^EPG: eggs per gram

^c^LPG: larvae per gram

^d^The presence of *Elaphostrongylus* *alces* and *Varestrongylus* *alces* larvae are reported, but larval counts are combined as protostrongylid-type larvae due to the presence of ambiguous individuals

### Metabarcoding assays

Amplicon sequencing generated a total of 6,053,739 high-quality sequences, with a mean of 65,775 per sample (range: 1796–1,234,331), of which 5,011,920 were assigned to the phylum Nematoda (mean: 57,608 sequences per sample, range: 16–276,841). Diverse GIN species assemblages were recovered from all moose faecal samples, including 10 genera from six strongylid families (*Chabertia*, *Cooperia*, *Elaphostrongylus*, *Haemonchus*, *Nematodirus*, *Ostertagia*, *Spiculopteragia*, *Teladorsagia*, *Trichostrongylus*, and *Varestrongylus*) (Fig. 3; Additional file 1: Figs. S1–S2). Nematode sequences were not recovered from the isolation and PCR-negative control samples. An average of 18.5 ASVs (sd: 6.7, range: 1–37) were recovered per moose individual, representing an average of 6.7 species (sd: 3.17, range: 1–13). Although the number of ASVs recovered was significantly correlated with sequencing depth, the number of species recovered was not, indicating that the sequencing depth was sufficient to recover all of the GIN species present in the samples (Additional file 1: Fig. S3, Additional file 2: Table S5). The most frequently occurring species belonged to *Ostertagia,* *Trichostrongylus*, and *Nematodirus* (Additional file 1: Figs. S1–S2). Compared with traditional parasitological investigations of the faecal samples, metabarcoding recovered a greater diversity of parasitic GIN families and genera (Fig. 3a) and provided higher taxonomic resolution for more of the occurrences detected (Fig. 3b).

**Fig. 4:**
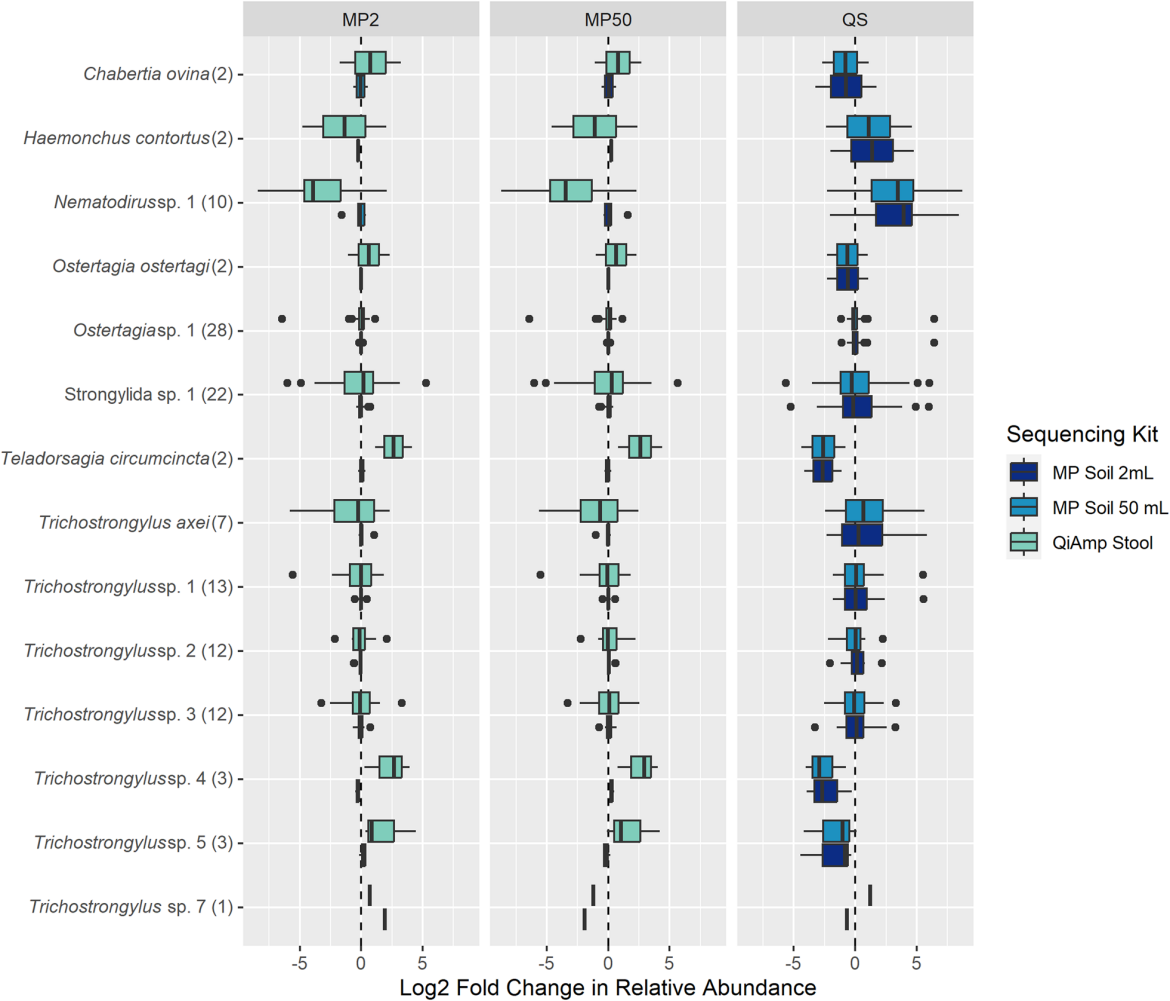
Differences in proportional abundance estimates across taxa. Each panel represents log fold change comparisons calculated using the method indicated in the panel label as a reference value and represented by the dashed vertical line. The number of faecal samples for which the log fold changes were calculated is indicated in parentheses after the taxon name. Samples with non-detection of a taxon by one or more methods were excluded from the analysis. *MP2* MP soil kit 2 ml, *MP50* MP soil kit 50 ml, *QS* Qiagen stool kit

**Fig. 5:**
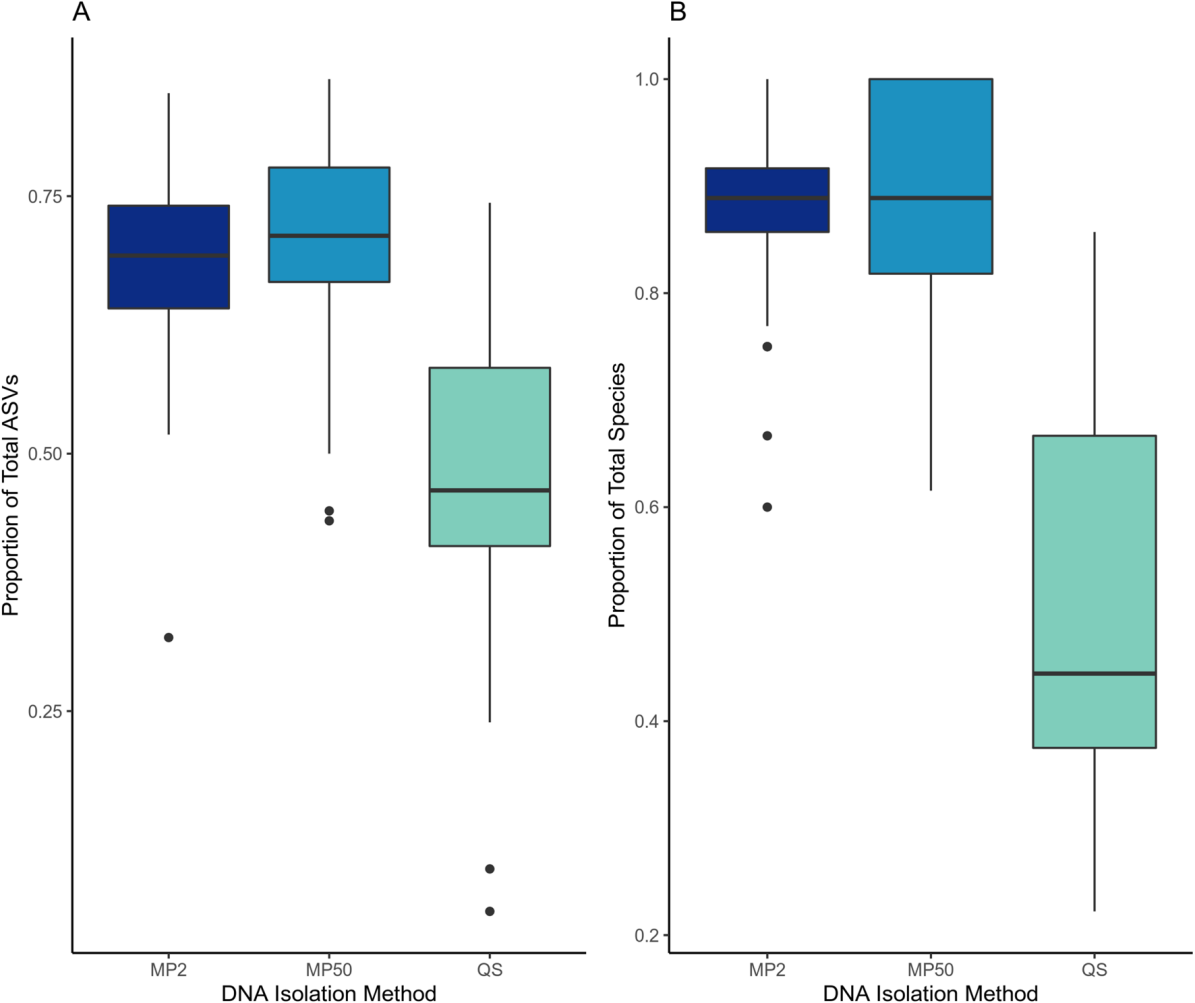
ASV and species recovery using different DNA isolation methods. Comparison of ASV (**a**) and species (**b**) recovery using different DNA isolation methods. Values represent the proportion of the total ASVs or species recovered per individual. MP2: MP Biomedicals FastDNA™ Spin Kit for Soil (2 ml), MP50: MP Biomedicals FastDNA™ Spin Kit for Soil (50 ml), QS: Qiagen QIAamp Fast DNA Stool Mini Kit

**Fig. 6:**
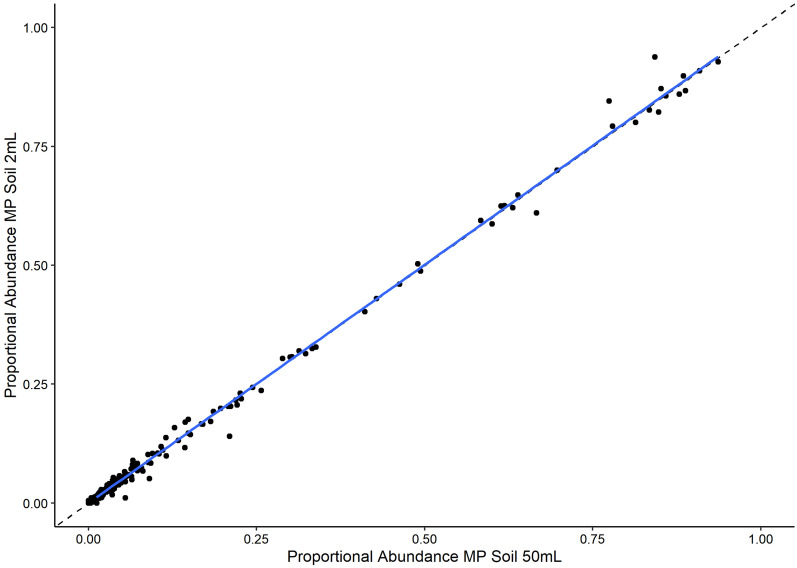
Comparison of bulk and sub-sampled DNA isolations. Correlation between the proportional abundance of GIN species when isolated from bulk faecal samples (MP Soil 50 ml) or from an aliquot of the same homogenized faecal sample (MP Soil 2 ml) (*P* < 2e−16, *R*^2^ = 0.997, *t* = 762.564). A 1:1 relationship is indicated by the dotted line, while the fitted correlation is indicated by a solid blue line

The three methods of DNA isolation tested recovered highly similar GIN communities from each individual with regards to composition (Additional file 1: Figs. S1–S2). The proportional abundances of individual taxa were consistent across methods for highly abundant taxa like *Ostertagia* sp. 1 and *Trichostrongylus* sp. 1, and more variable among low abundance taxa like *Nematodirus* sp. 1 and *Trichostrongylus* *axei* (Fig. 4). Proportional abundances were more consistent between the MP soil kit extractions than between either the MP soil kit or the QIAamp stool kit (Fig. 4). Among taxa with > 10 comparisons, we did not observe consistent, systematic over or under estimation by any of the methods (Fig. 4). The Qiagen stool kit-based protocol (220 mg starting material) yielded significantly fewer ASVs (degrees of freedom [*df*] = 86, t = −6.228, *P* < 0.001) and species (*df* = 86, *t* = −10.026, *P* < 0.001) than the MP soil kit-based protocols (2–4 g starting material, Fig. 5). There were no significant differences in ASV and species recovery between the 2 ml and 50 ml MP soil kit protocols (ASVs: *df* = 86, *t* = 0.468, *P* = 0.641; species: *df* = 86, *t* = 0.036, *P* = 0.971) (Fig. 5). In addition, there was a strong correlation between the proportional abundances of species recovered from the bulk isolation and the 2 ml aliquot for any given sample (*df* = 1536, *t* = 762.564, *P* < 2e−16, Fig. 6), highlighting the consistency of results when using either of the MP soil kit protocols. Although ASV recovery was significantly correlated with sequencing depth, species recovery was not, and there was no significant interaction between isolation protocol and sequencing depth (Additional file 1: Fig. S2, Additional file 2: Table S5).

**Fig. 7:**
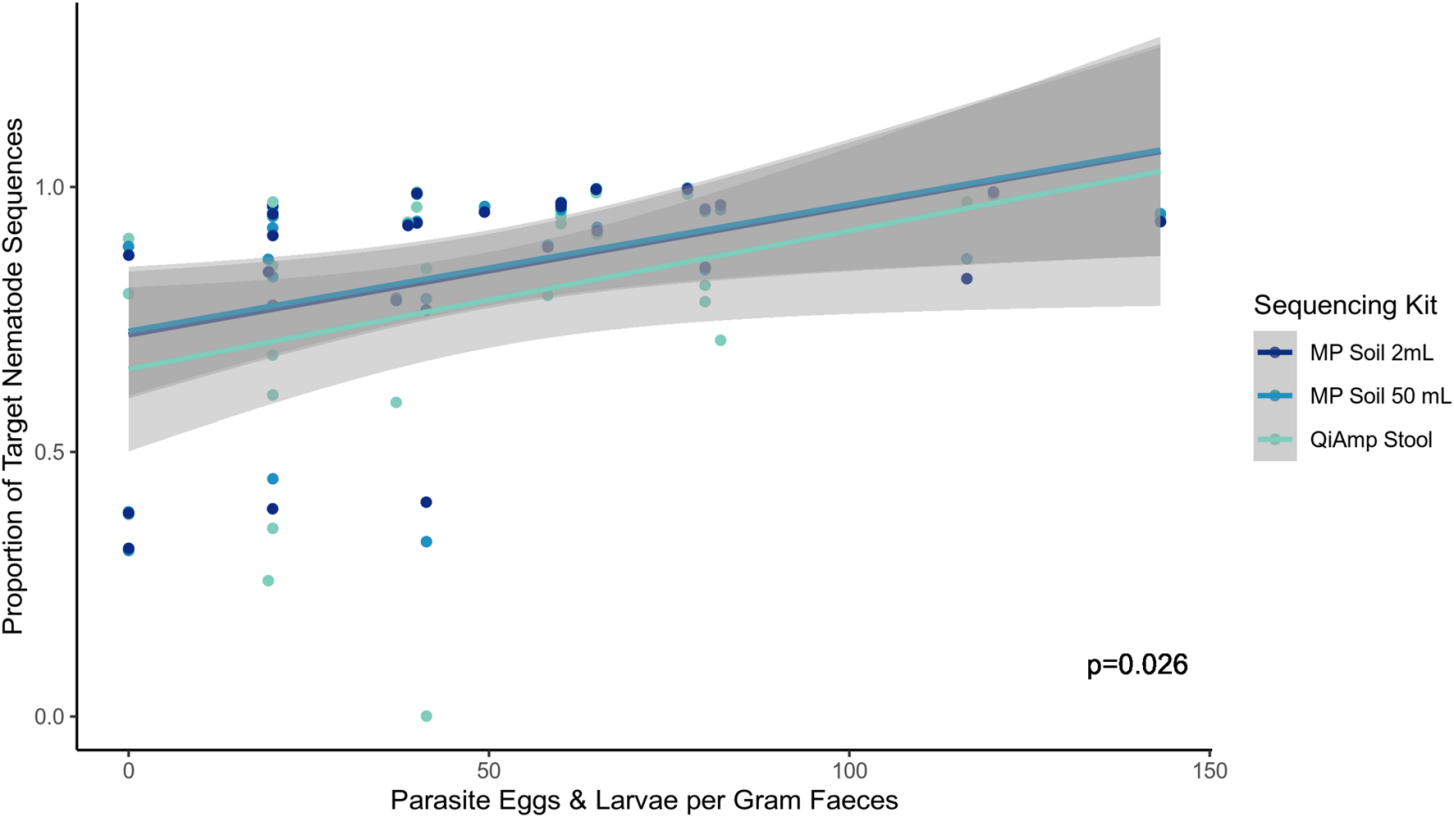
Parasite load in 29 moose estimated by molecular and parasitological assays of faecal samples. The parasite load of moose was estimated from metabarcoding data as the proportion of nematode to non-target sequences recovered and then compared with parasitological egg and larvae counts (*df* = 86, *t* = 2.445, *P* = 0.026). Results are shown for each of the three different DNA extraction methods tested

The GIN communities recovered by metabarcoding from the faeces were largely consistent with those recovered using traditional parasitological investigations, albeit with higher taxonomic resolution (Fig. 3; Additional File 1: Fig. S1). However, the results were not entirely congruent, as metabarcoding detected *E. alces* and *V. alces* in only two of the four samples where they were recovered by parasitological investigations (Additional file 1: Fig. S1). Furthermore, metabarcoding recovered GIN species from two samples where no eggs or larvae were observed during the parasitological investigations (Additional file 1: Fig. S1). The proportion of metabarcoding reads that could be assigned to target nematode taxa varied greatly between samples (range: 0.08–99.8%) and was significantly correlated with the individual’s total egg and larval load (*df* = 86, *t* = 2.445, *P* = 0.026) (Fig. 7).

## Discussion

Although wild ungulate populations are heavily monitored throughout Europe, we understand little of how parasites affect population dynamics, and there is no systematic, long-term monitoring of parasite diversity and parasite loads. Such monitoring is in part hampered by lack of time- and cost- effective assay methodologies with high sensitivity and good taxonomic resolution. DNA-based methods are increasingly used for the characterization of biodiversity in a variety of contexts [46], and here we explore the suitability of DNA metabarcoding for parasite monitoring and attempt to optimize the DNA isolation step of this method.

### Effects of DNA isolation method

Both ASV and species recovery was higher when DNA was isolated with the MP soil kit as compared with the Qiagen stool kit, indicating that GIN assay sensitivity and resolution can be substantially impacted by the DNA isolation method. The importance of DNA isolation in the detection of parasitic nematode species has been highlighted during the development of diagnostic quantitative PCR (qPCR) tests for commercially relevant species [34, 47, 48]. The eggs of GIN species are known to be recalcitrant and difficult to break open [47], which can prevent effective DNA isolation. The MP soil kit includes a mechanical grinding step intended to physically disrupt cells, while the Qiagen stool kit does not and instead depends only on chemical lysis to free cellular DNA within the sample. It would appear the grinding step in the MP soil kit successfully ruptured more nematode eggs in the samples and a physical homogenization step is important for optimizing the sensitivity of metabarcoding assays for GIN communities. However, it must also be noted that the total starting faecal biomass used in the Qiagen stool kit was approximately 220 mg, while the MP soil kit used an order of magnitude more starting biomass (2–4 g). The increased starting material effectively increases the sampling effort, which, as would be expected, yields greater sensitivity in the assays. The lack of significant differences in ASV and species recovery and strong correlation in species proportional abundances between the isolation from a 2-ml aliquot of the homogenized faecal material and the entire biomass with the MP soil kit suggests that the time- and cost-saving advantages of a 2 ml-based extraction protocol can be retained without sacrificing metabarcoding assay sensitivity, as long as there is a preliminary homogenization step with larger amounts of faecal biomass. Use of a 2 ml-based extraction kit allows simultaneous treatment of 24–96 samples at all steps of the isolation protocol, while 50-ml bulk extractions are restricted to simultaneous handling of eight samples in some steps of the isolation protocol. In addition, there was a 60% cost saving per sample in using the described sub-sampling method with a 2-ml kit as opposed to doing bulk isolations. Simplification and streamlining of laboratory protocols for DNA extraction and metabarcoding contribute to reducing costs and increasing time efficiency, further increasing the utility of non-invasive metabarcoding for large scale monitoring of GIN communities in wild populations.

### Metabarcoding for characterizing GIN communities in wild ungulates

DNA-based methods are increasingly used for the characterization of biodiversity in a variety of contexts [46], and in general, have proven to be both more sensitive and provide better taxonomic resolution for the taxa detected [49]. In this paper, we demonstrate that DNA metabarcoding is a highly valuable approach for the characterization of GIN parasites in wild ungulates such as moose. Using a molecular-based approach, we detected GIN species in all samples investigated, while egg and larval counts detected GIN in 93% of samples. Nevertheless, *E. alces* and *V. alces* were detected exclusively by morphological assays in two of the samples. These apparent detection failures by the metabarcoding method could be a result of primer-related bias, although this seems unlikely given that both species were successfully detected in other samples. Instead, the differences in the detection of *E. alces* and *V. alces* between the methods may be attributed to stochastic differences in the faecal subsamples subjected to parasitological and metabarcoding analyses, as different volumes of faecal matter were analysed, and eggs and larvae can be unevenly distributed between faecal pellets. These stochastic differences in the occurrence of eggs and larvae in the faecal material may also be driving the increased GIN detection rate observed with the metabarcoding approach. Alternatively, the increased detection rate could be due to contamination or false positives using the metabarcoding method, although we argue this is unlikely, as there was no systematic contamination observed in the sequenced PCR and extraction negative controls, and multiple species were detected in each of the samples. Given that no GIN taxa were detected solely by parasitological methods and not concurrently by metabarcoding, we instead argue that metabarcoding of GIN DNA isolated from frozen faecal samples has increased sensitivity when compared with egg and larval counts from the same samples when they are fresh, most likely in cases with low egg and larval abundance. Other PCR-based methods have been demonstrated to have increased sensitivity over traditional microscopy-based methods for GIN detection [50–53], but to our knowledge, this has not been previously demonstrated for DNA metabarcoding. We hypothesize that this increased sensitivity can be attributed to the metabarcoding method also detecting extracellular DNA derived from adult worms [54] in the gastrointestinal tract that may be shedding few or no eggs at the time of sampling. While other species-specific PCR-based methods may have similar detection sensitivity with better cost-effectivity, DNA metabarcoding-based approaches do not require a priori knowledge of the GIN community and have the potential to detect unexpected and/or atypical GIN infections.

The metabarcoding approach consistently recovered more GIN genera and families, providing improved taxonomic resolution as compared with traditional morphological assays. This is primarily driven by the capacity for metabarcoding methods to distinguish between strongyle-type eggs that cannot be identified to species based on morphology [17]. Only three GIN genera were detected with traditional methods as compared with 10 using DNA metabarcoding. Such improvement in taxonomic resolution allows for better estimation of the diversity and the range of species infecting a given individual. Although the metabarcoding approach improved taxonomic resolution over the morphological assays, it must be noted that the primer combination used (NC1–NC2) is limited to clade V GIN, and as such will not detect other parasite groups that are typically included in Baermann and McMaster assays (e.g. *Moniezia, Eimeria*, *Trichuris*, *Capillaria*). Moreover, several of the GIN sequence variants recovered could only be identified with high confidence to the genus, family, or order level. Of the 10 most abundant ASVs identified to these higher taxonomic levels, six had 98% identity or less to a reference sequence in the database, suggesting that there is a lack of reference sequences for GIN parasites of wild ungulates. For example, *Spiculopteragia alcis* and *Ostertagia kolchida* are two known GIN parasites of moose that were not included in the identification database. Further reference database development will be needed to support the implementation of DNA metabarcoding in large-scale monitoring of GIN infections in these wild populations.

Finally, measures of parasite load are of particular interest for monitoring GIN infections, as they correlate with host body condition, fecundity, and survival in populations of wild ungulates [55, 56]. Traditional egg and larval count methods from faecal samples provide a non-invasive method for estimating parasite load, but involve laborious isolation procedures that make the method suboptimal for large-scale monitoring programs where high throughput of many samples is required. In the current study, we observe a significant relationship between the proportion of nematode sequences recovered from the samples and the parasite load as determined by egg and larval counting. On the individual species level, it is well documented that DNA metabarcoding sequence abundance is at best, semi-quantitative [57] in relation to the number of individuals or biomass, although the method provides robust estimates of proportional abundances within GIN communities in a single host [58]. The correlation between total parasite load and the ratio of nematode sequences to non-target sequences has not previously been reported. While DNA metabarcoding may be unreliable for estimating individual species abundances, this result suggests it may nevertheless provide a very coarse estimate of the total parasite load. However, this result must be interpreted with extreme caution given the small number of samples (*n* = 29), and the small number of samples with high parasite load (> 100 eggs and larvae per gram: three samples). Further research is required to determine whether this relationship can provide a meaningful index for parasite loads at levels affecting host condition, which would be relevant for management and conservation in wild populations.

### Host specificity of parasites and spillover among host species

Wild ungulates can act as infection reservoirs for domestic hosts [30, 59]. With evidence that parasite loads in wild ungulate populations are affected by land use (specifically livestock rearing) and climate change [60, 61], a better understanding of the dynamics of host-parasite interactions and the ensuing effects on host population dynamics is urgent. A major benefit of the metabarcoding approach is increased taxonomic resolution. Such insight is required to understand the host specificity of the parasite community and to predict the parasite spillover in host communities of wild and domestic ungulates. A number of the GIN species detected in the moose faecal samples are commonly known from domestic animals (e.g. *Chabertia ovina*, *Cooperia oncophora*, and *Teladorsagia circumcincta*) where they cause host morbidity [17]. This is consistent with earlier observations that GIN taxa in co-occurring wild cervids and domestic animals frequently overlap [62–65] and further supports the theory that wild ungulate populations can act as reservoirs for GIN parasites of domestic animals with reciprocal infections occurring between species [59, 62]. The high taxonomic resolution of DNA metabarcoding-based GIN monitoring in wild ungulate populations has the potential to provide not only valuable data for conservation and management decisions, but also provide insight into the parasite spillover between co-occurring wild and domestic species and their impact on each other’s health.

## Conclusions

DNA metabarcoding is a promising technique for the non-invasive, large-scale monitoring of parasitic GINs in wild ungulate populations. Metabarcoding assays provide increased sensitivity and taxonomic resolution compared with traditional egg and larva isolation and identification methods. While not strictly a quantitative method, our results indicate that with further research, it may nonetheless be possible to create a management- and conservation-relevant index for host parasite load. The DNA isolation method significantly impacted species recovery, and for monitoring of GIN species from faecal samples, we recommend the use of a DNA isolation protocol that (1) includes a mechanical cell disruption step and (2) maximizes starting material volume.

## Supplementary Information

**Additional file 1: Figure S1.** Comparison of 29 moose individuals’ parasitic nematode communities detected using parasitological and metabarcoding assays of faecal samples. Parasitological surveys included counting of eggs and larvae. Metabarcoding of faeces samples was conducted using three different DNA isolation protocols. Point type indicates the lowest taxonomic level a method successfully identified the taxon at. **Figure S2.** Taxonomic summary of the gastrointestinal nematode community recovered by DNA metabarcoding. Faecal samples of 29 moose were analysed. Sequences that could not be identified to the species level are grouped at the lowest level of taxonomy possible, and the number of operational taxonomic units (OTUs) recovered in the group is indicated in parentheses following the taxon name. **Figure S3.** The relationship between sequencing depth and the total number of (A) ASVs and (B) species recovered.

**Additional file 2: Table S1.** Detection of parasitic gastrointestinal nematode eggs in moose faecal samples using the McMaster technique. **Table S2.** Quantification of parasitic gastrointestinal nematode eggs in moose faecal samples using the McMaster technique. **Table S3.** Detection of parasitic gastrointestinal nematode larvae in moose faecal samples using the Baermann technique. **Table S4.** Quantification of parasitic gastrointestinal nematode larvae in moose faecal samples using the Baermann technique. **Table S5.** Model results from general linear models assessing the relationship between sequencing depth and isolation method and the number of ASVs and species recovered.

## Data Availability

All data generated or analysed during this study are included in this published article and its supplementary information files or are deposited in the NCBI SRA repository under the accession number PRJNA856286.

## Abbreviations

DNA: Deoxyribonucleic acid
GIN: Gastrointestinal nematode
ITS: Internal transcribed spacer
PCR: Polymerase chain reaction
