# Supplementary material for: Faecal metabarcoding provides improved detection and taxonomic resolution for non-invasive monitoring of gastrointestinal nematode parasites in wild moose populations
Source: Parasit Vectors. 2023 Jan 18;16:19. doi: 10.1186/s13071-022-05644-6 (PMC9847159; doi:10.1186/s13071-022-05644-6)

Supplementary Figures

Figure S1. Comparison of 29 moose individuals' parasitic nematode communities detected using parasitological and metabarcoding assays of faecal samples. Parasitological surveys included counting of eggs and larvae. Metabarcoding of faeces samples was conducted using three different DNA isolation protocols. Point type indicates the lowest taxonomic level a method successfully identified the taxon at.

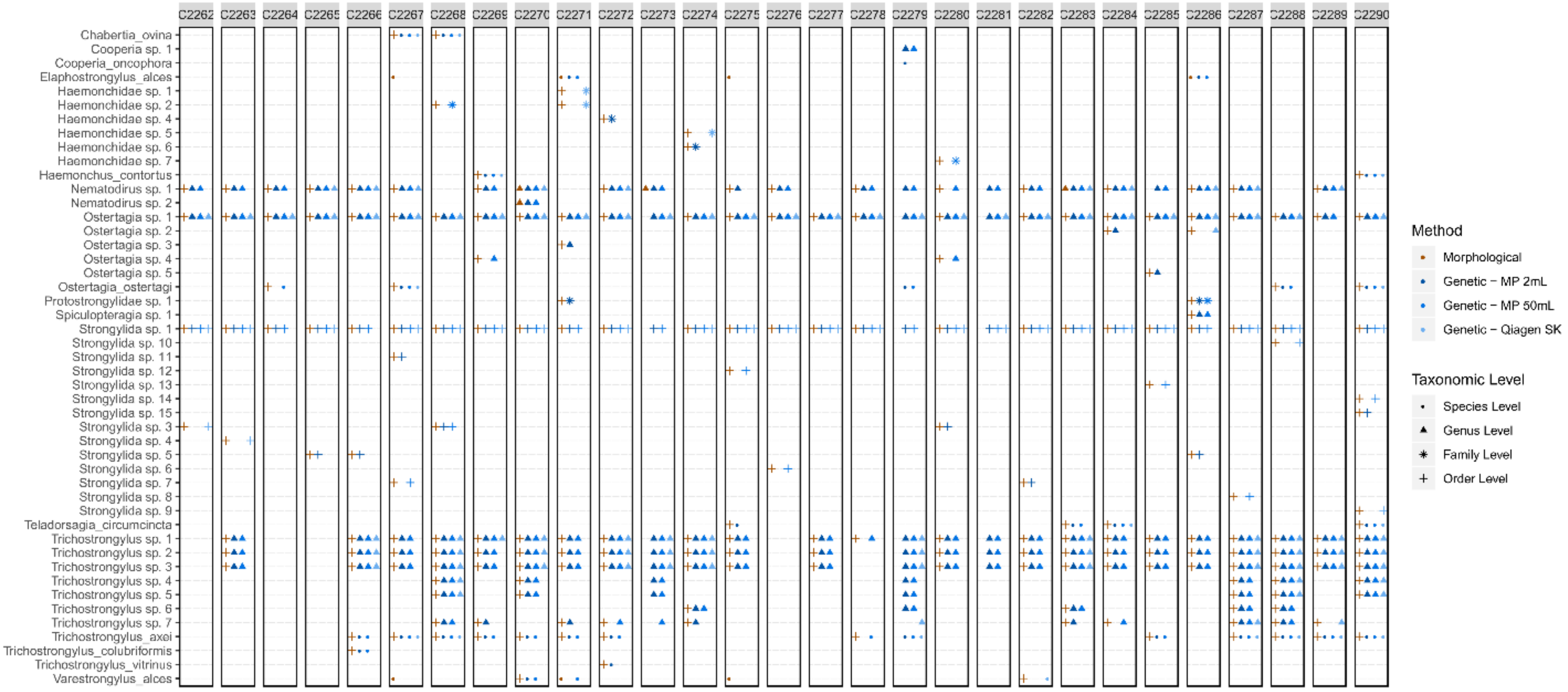

Figure S2. Taxonomic summary of the gastrointestinal nematode community recovered by DNA metabarcoding. Faecal samples of 29 moose were analysed. Sequences that could not be identified to the species level are grouped at the lowest level of taxonomy possible, and the number of operational taxonomic units (OTUs) recovered in the group is indicated in parentheses following the taxon name.

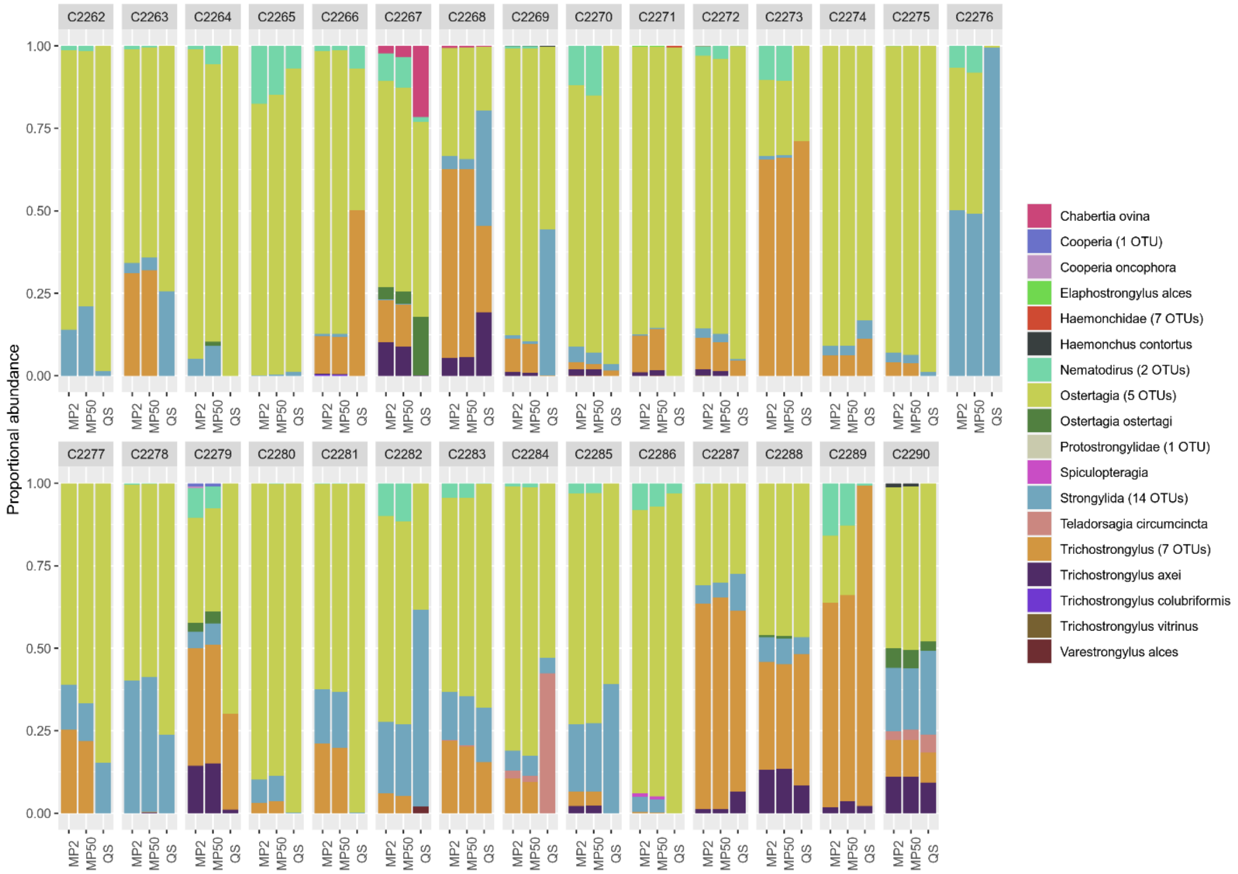

Figure S3. The relationship between sequencing depth and the total number of (A) ASVs and (B) species recovered.

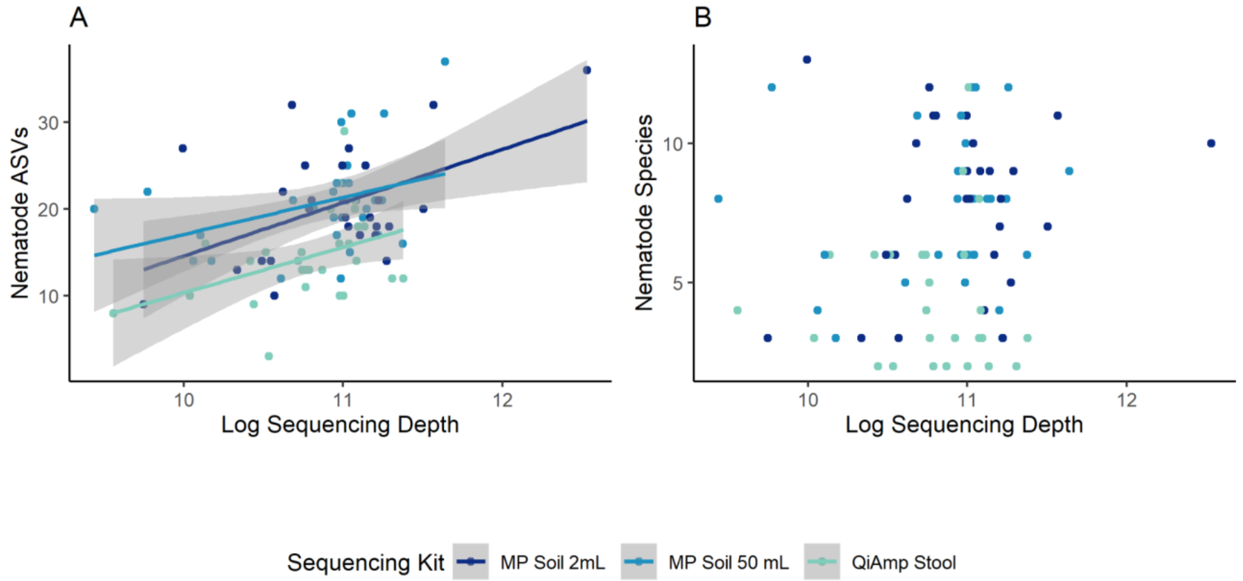

Supplement: Supplementary file 1 — Additional file 1: Figure S1. Comparison of 29 moose individuals’ parasitic nematode communities detected using parasitological and metabarcoding assays of faecal samples. Parasitological surveys included counting of eggs and larvae. Metabarcoding of faeces samples was conducted using three different DNA isolation protocols. Point type indicates the lowest taxonomic level a method successfully identified the taxon at. Figure S2. Taxonomic summary of the gastrointestinal nematode community recovered by DNA metabarcoding. Faecal samples of 29 moose were analysed. Sequences that could not be identified to the species level are grouped at the lowest level of taxonomy possible, and the number of operational taxonomic units (OTUs) recovered in the group is indicated in parentheses following the taxon name. Figure S3. The relationship between sequencing depth and the total number of (A) ASVs and (B) species recovered. [file 13071_2022_5644_MOESM1_ESM.pdf]
